# Transmission Incompetence of *Culex quinquefasciatus* and *Culex pipiens pipiens* from North America for Zika Virus

**DOI:** 10.4269/ajtmh.16-0865

**Published:** 2017-05-03

**Authors:** Joan L. Kenney, Hannah Romo, Nisha K. Duggal, Wen-Pin Tzeng, Kristen L. Burkhalter, Aaron C. Brault, Harry M. Savage

**Affiliations:** 1 Centers for Disease Control and Prevention, Fort Collins, Colorado

## Abstract

In late 2014, Zika virus (ZIKV; *Flaviviridae*, *Flavivirus*) emerged as a significant arboviral disease threat in the Western hemisphere. *Aedes aegypti* and *Aedes albopictus* have been considered the principal vectors of ZIKV in the New World due to viral isolation frequency and vector competence assessments. Limited reports of *Culex* transmission potential have highlighted the need for additional vector competence assessments of North American *Culex* species. Accordingly, North American *Culex pipiens* and *Culex quinquefasciatus* were orally exposed and intrathoracically inoculated with the African prototype ZIKV strain and currently circulating Asian lineage ZIKV strains to assess infection, dissemination, and transmission potential. Results indicated that these two North American *Culex* mosquito species were highly refractory to oral infection with no dissemination or transmission observed with any ZIKV strains assessed. Furthermore, both *Culex* mosquito species intrathoracically inoculated with either Asian or African lineage ZIKVs failed to expectorate virus in saliva. These in vivo results were further supported by the observation that multiple mosquito cell lines of *Culex* species origin demonstrated significant growth restriction of ZIKV strains compared with *Aedes*-derived cell lines. In summation, no evidence for the potential of *Cx. pipiens* or *Cx. quinquefasciatus* to serve as a competent vector for ZIKV transmission in North America was observed.

## Introduction

Zika virus (ZIKV), a member of the genus *Flavivirus*, has recently surfaced as a significant threat to global public health despite only having been found in the Western hemisphere since late 2014. The first ZIKV isolates were made from a sentinel Rhesus monkey and subsequently from arboreal *Aedes africanus* mosquitoes, a suspected sylvatic cycle vector,^1^ in Uganda in 1947 and 1948.^2^ The first described clinical case was identified in Nigeria in 1954 in which the individual exhibited symptoms similar to many arthralgic arboviruses including fever, headache, diffuse joint pain, and slight jaundice.^3^ Serological evidence of ZIKV infection was subsequently identified in Nigeria, Egypt, India, Malaysia, Indonesia, Philippines, Thailand, Vietnam, and Senegal.^4^–^11^ Phylogenetic analysis demonstrates three distinct ZIKV genotypes corresponding to the initial emergence in east Africa and subsequent spread to west Africa and Asia.^12^ ZIKV was associated with sporadic limited human disease prior to 2007 when ZIKV caused outbreaks in Yap and Gabon.^13^,^14^ By 2013, ZIKV had spread to French Polynesia and other islands of the South Pacific including New Caledonia, the Cook Islands, and Easter Island prior to its first detection in the Western hemisphere in 2014.^15^

First detected in the peridomestic vector, *Aedes aegypti*, in Malaysia in 1966,^16^ subsequent vector competence and ecological studies have established ZIKV to be vectored primarily by members of the genus *Aedes* in both sylvatic and urban cycles through field studies.^2^,^16^–^24^ However, recent ZIKV detection in *Culex* mosquitoes^25^,^26^ and reports of experimental evidence of transmission of ZIKV by *Culex quinquefasciatus*,^26^,^27^ have raised public concerns that mosquitoes of the *Culex pipiens* complex could play a role in transmission as well. Vector competence by mosquitoes of alternative genera would have possible implications for many regions of North America where the known *Aedes* vectors, *Ae. aegypti* and *Aedes albopictus*, are absent and a significant number of *Culex* species are present. The utilization of both *Aedes* and *Culex* mosquitoes as vectors would alter the discourse and strategy for control efforts. Surveillance and control of *Culex* mosquitoes requires utilization of different tools and greatly increases the area of surveillance based on the wide geographic range of potential *Culex* vectors.

There have been a number of recent studies directly evaluating the vector competence of *Culex* mosquitoes for circulating strains of ZIKV and the results have been mixed. Two studies using *Cx. pipiens* and *Cx. quinquefasciatus* from North America showed a lack of ZIKV infection, dissemination, or saliva infection after oral exposure by artificial or viremic mice as blood meal sources.^28^,^29^ A study from Brazil showed a low level of experimental infection in *Cx. quinquefasciatus* after artificial blood meal exposure, but no dissemination from the midgut or transmission.^30^ An Italian study indicated a lack of infection in *Cx. pipiens* mosquitoes after oral exposure,^31^ whereas a second study in Europe examining ZIKV oral infection of *Cx. pipiens* and *Cx. quinquefasciatus* found evidence of infection and low rates of dissemination but no transmission.^32^ The findings from experiments described above are in direct contrast with two reports of highly efficient transmission potential from Brazil^26^ and China.^27^ In an attempt to rule out the role of *Culex* mosquitoes as possible ZIKV vectors in North America, we evaluated *Cx. quinquefasciatus* and *Cx. pipiens* mosquitoes for susceptibility to infection after oral and intrathoracic (IT) exposure to recent Asian genotype ZIKV isolates from Puerto Rico and Honduras as well as the original African lineage isolate from Uganda. Additionally, we compared the growth capacity of each virus in model *Aedes* and *Culex* cell lines to highlight the lack of replication competence of ZIKV for in vitro and in vivo *Culex* models.

## Materials and Methods

### Viruses and mosquitoes.

ZIKV isolates MR766 (Uganda 1947), PRVABC59 (Puerto Rico 2015), and R103451 (Honduras 2016) were used for mosquito infections in this study. The MR766 strain had a history of 149 passages in unknown sources, including suckling mouse brain, and two known passages in African green monkey kidney (Vero) epithelial cells. PRVABC59 was isolated from the serum of a febrile traveler returning to the continental United States from Puerto Rico in 2015 and was passaged three times in Vero cells.^33^ The R103451 isolate was derived from a human placenta from a patient who had traveled to Honduras in 2015 and passaged in C6/36 mosquito cells once. Colonized *Cx. quinquefasciatus* (Sebring) and *Cx. pipiens pipiens* (Chicago) were used for in vivo exposure experiments. The colony of *Cx. quinquefasciatus* was originally established in Florida in 1988 and has since been maintained at the Centers for Disease Control and Prevention in Fort Collins, CO, since 2004.^34^ The original *Cx. pipiens* were collected in Chicago as mated, overwintering females in 2010 and distinguished from *Cx. pipiens* form molestus through microsatellite testing.^35^

### Oral mosquito infections.

For each exposure dose of each virus, cohorts of 5- to 6-day-old female *Cx. quinquefasciatus* and *Cx. pipiens* were orally exposed to individual ZIKV strain containing blood meals through an artificial Hemotek membrane feeder (Discovery Workshops, Accrington, United Kingdom). Frozen stocks of known titers were thawed and used for blood meal exposure. For *Cx. quinquefasciatus*, two cohorts of 50 adult females were exposed to the same dose of MR766 (6.0 log_10_ plaque-forming units [PFU]/mL); three cohorts were exposed to 4.0, 5.9, and 7.1 log_10_ (PFU/mL) of PRVABC59; and two cohorts were exposed to 6.4 and 7.6 log_10_ (PFU/mL) of R103451. For orally exposed *Cx. pipiens*, one cohort each was exposed to 6.0 log_10_ (PFU/mL) of MR766 and PRVABC59. Female mosquitoes were allowed to feed for 45 minutes on the heated artificial blood meals containing 33% (v/v) defibrinated goose (*Cx. pipiens*) or calf (*Cx. quinquefasciatus*) washed erythrocytes (Colorado Serum Company, Denver, CO), 33% (v/v) heat-inactivated fetal bovine serum (FBS) (Omega Scientific, Inc., Tarzana, CA), 33% (v/v) of each individual virus in cell culture fluid (resulting in a final concentration ranging from 4.0 to 7.1 log_10_ PFU/mL) and 1% (v/v) of 0.25 μM adenosine triphosphate (Table 1). After blood feeding, mosquitoes were cold-anesthetized for sorting, and engorged females were held at 28°C with a relative humidity of 70–75% for an extrinsic incubation period of 14 days.

### Mosquito processing and testing.

At the conclusion of the 14-day extrinsic incubation period, mosquitoes were anesthetized with triethylamine (Flynap^®^; Carolina Biological Supply company, Burlington, NC) as previously described.^36^ Salivations were performed by insertion of the proboscis of each immobilized mosquito into a 10-μL capillary tube containing immersion oil (Cargille Laboratories, Cedar Grove, NJ) to induce salivation for approximately 45 minutes. Legs and wings were removed from anesthetized mosquitoes and placed in microcentrifuge tubes with 500 μL of complete media composed of Dulbecco's modified Eagle's essential medium (DMEM) complete with penicillin (100 U/mL), streptomycin (100 mg/mL), 10% FBS, amphotericin B (50μg/mL), and a sterile copper bead. After salivation, bodies and salivary capillary tubes were separated into 1.5-mL tubes with 500 μL and 350 μL of complete media, respectively. Samples with mosquito bodies and legs/wings were each triturated for 4 minutes at a frequency of 26 cycles per second using a Mixer Mill 300 (Retsch, Newton, PA). Samples containing salivary capillary tubes were centrifuged to clarify supernatants. Each sample was tested for cytopathic effects (CPEs) on Vero cells as previously described.^37^ Collected salivary material for a given sample was not tested if dissemination was not detected.

### IT mosquito infections.

Cohorts of 50 adult female *Cx. quinquefasciatus*, *Cx. pipiens*, and *Ae. aegypti* colonized from Poza Rica, Mexico,^38^ were subjected to IT inoculation of approximately 300 PFU (0.33 μL inoculation of 6 log_10_ [PFU/mL]) of PRVABC59 or MR766 ZIKV. Mosquitoes were held for an extrinsic incubation period of 7 days at 28°C with a relative humidity of 70–75%. Mosquitoes were processed as described above. After processing, bodies and saliva samples were stored at −80°C until titration for virus by plaque assay or viral RNA testing. CPE-negative saliva from intrathoracically inoculated mosquitoes was subsequently analyzed by real-time reverse transcription polymerase chain reaction (qRT-PCR)^39^ to confirm negative results. Viral RNA was extracted using the QIAamp Viral RNA Mini Kit (Qiagen, Valencia, CA) according to the manufacturer's protocol.

### In vitro growth on *Culex* cell lines.

Replication profiles were assessed on *Ae. aegypti* (Aag-2), *Ae. albopictus* (C6/36), *Cx. quinquefasciatus* cells (Hsu), and *Culex tarsalis* (CT)–derived cell lines. All cell lines were propagated in DMEM supplemented with 5% FBS, penicillin (100 U/mL), and streptomycin (100 mg/mL). Cells were grown to 90% confluency in 6-well plates, and monolayers were infected in triplicate at a multiplicity of infection (MOI) of 0.1 PFU/cell. 50-μL aliquots of supernatant were collected daily, followed by plaque assays to measure viral yield. Plaque assays used serial 10-fold dilutions of virus seeded onto 6-well plates containing monolayers of Vero cells, as previously described.^37^ To compare the viral replication curves, a two-way analysis of variance (ANOVA) test with repeated measures and post hoc multiple comparisons test with a Tukey correction was performed, version 6.0h (Prism Graphpad Software, La Jolla, CA). *P* values < 0.05 were considered significant.

## Results

### Oral infection, dissemination, and transmission in *Culex* mosquitoes.

Oral infection rates were low for both *Cx. quinquefasciatus* (0–1%) and *Cx. pipiens* (1–10%) regardless of the virus used (Table 1). For example, only 1/95 *Cx. quinquefasciatus* was identified to be infected after oral exposure with 6.0 log_10_ PFU/mL of MR766. Similarly, no infected *Cx. quinquefasciatus* mosquitoes were observed after oral exposure to PRVABC59 with doses ranging from 4.0 to 7.1 log_10_ (PFU/mL) or to R103451 with doses of either 6.4 or 7.6 log_10_ (PFU/mL) (Table 1). There was no detectable dissemination of virus from the midgut of the single *Cx. quinquefasciatus* mosquito body identified to be ZIKV positive after oral exposure to MR766 as determined by CPE testing of legs/wings. For *Cx. pipiens*, 1/20 (5%) and 4/38 (11%) were found to have detectable virus in their bodies after exposure to 6.0 log_10_ (PFU/mL) of MR766 or PRVABC59, respectively (Table 1). It should be noted that the four positive *Cx. pipiens* mosquitoes only had one plaque per well after undiluted inoculation of Vero cells, indicating an approximate concentration of 3 PFU/mosquito body. None of these *Cx. pipiens* were identified to have a disseminated infection as defined by the presence of virus in legs/wings.

### Infection and transmission of inoculated mosquitoes.

To determine whether *Cx. pipiens* and *Cx. quinquefasciatus* have the capability to transmit regardless of their apparent inability to establish infection of the midgut and disseminate virus, IT inoculations were performed to bypass the midgut infection and escape barriers. This allowed for direct assessment of ZIKV to infect the salivary glands and subsequently be transmitted during salivation. Seven days after IT inoculation, 16/23 (70%) and 5/33 (15%) *Cx. quinquefasciatus* bodies were identified to be positive for MR766 and PRVABC59 viruses, respectively (Table 2). Plaque assay and subsequent qRT-PCR showed no virus or viral RNA–positive saliva samples. Experiments on *Cx. pipiens* provided similar results with 17/28 (61%) triturated bodies demonstrating virus 7 days after inoculation with PRVABC59 (Table 2), yet no evidence of transmissibility was observed by plaque assay or qRT-PCR assessments of expectorants. In contrast, evaluation of the IT susceptibility of *Ae. aegypti* showed that 12/12 (100%) of the surviving mosquitoes became infected after inoculation and 8/12 (67%) of infected mosquitoes demonstrated the presence of virus in salivary material indicating transmission potential.

### In vitro growth of ZIKV strains in *Aedes* and *Culex* mosquito cells.

**Figure 1.**
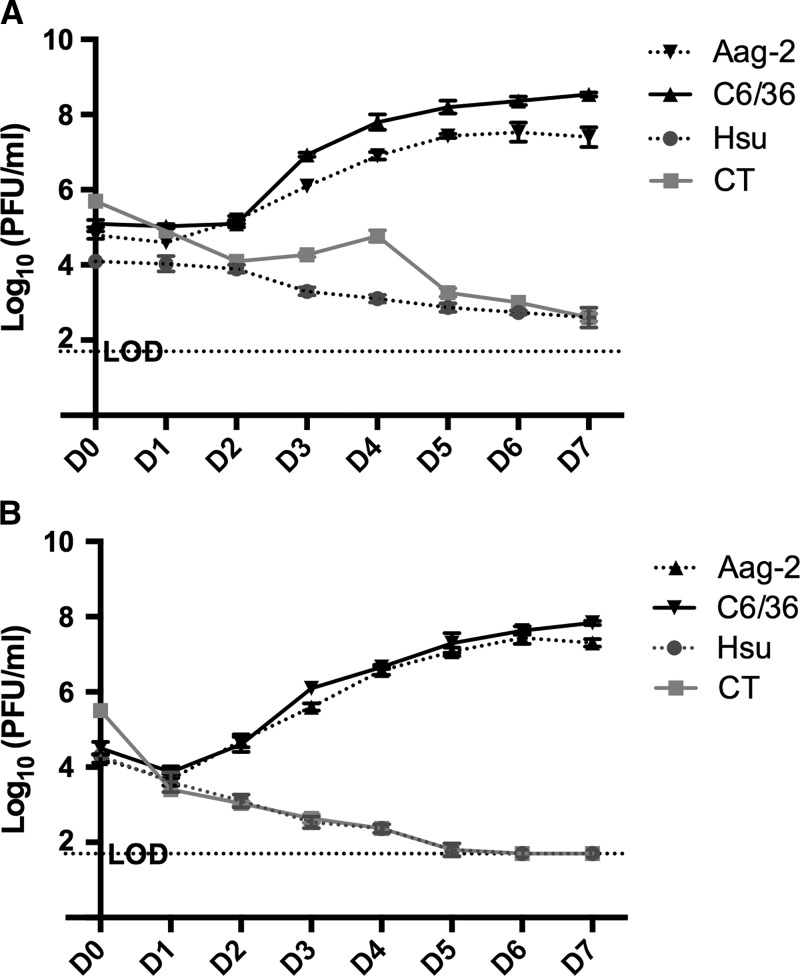
Zika virus (ZIKV) growth in mosquito cells. Comparison of (**A**) PRABCV59 and (**B**) MR766 replication in *Aedes aegypti* (Aag-2), *Aedes albopictus* (C6/36), *Culex quinquefasciatus* (Hsu), and *Culex tarsalis* (CT) cells. LOD = limit of detection. Error bars represent standard deviations.

*Culex quinquefasciatus*, *Cx. tarsalis*, *Ae. aegypti*, and *Ae. albopictus* cell lines were each inoculated at an MOI of 0.1 PFU/cell with PRVABC59 and MR766 and sampled daily for 7 days. A two-way repeated measures ANOVA for each virus demonstrated a significantly different level of growth between the three types of mosquito cells (*P* < 0.0001) (Figure 1

). PRVABC59 reached a mean peak titer of 8.5 log_10_ (PFU/mL) on day postinoculation (dpi) 7 and 7.5 log_10_ (PFU/mL) on dpi 6 for *Ae. albopictus* and *Ae. aegypti* cells, respectively. The Puerto Rico virus titer in both *Culex* cell lines was highest on the initial sampling point at 0 dpi and dropped over the time course, indicating a growth rate below the loss of viral infectivity in the cultures over time. As such, mean titers of PRVABC59 growth in *Ae. albopictus* cells and *Ae. aegypti* were significantly higher (*P* < 0.0001) than either *Culex* cell line (Figure 1A). Similarly, MR766 reached a mean peak titer of 7.8 log_10_ (PFU/mL) on dpi 7 and 7.4 log_10_ (PFU/mL) on dpi 6 in *Ae. albopictus* and *Ae. aegypti* cells. Subsequent multiple comparison testing of MR766 demonstrated growth reached significantly higher mean viral titers (*P* < 0.0001) on *Ae. albopictus* and *Ae. aegypti* cells as compared with each of the *Culex* cell lines, as viral titers continued to drop for these two cell lines from dpi 0–7 and never appeared to grow (Figure 1B).

## Discussion

The potential vector competence of *Cx. pipiens* and *Cx. quinquefasciatus* was evaluated after oral and IT exposure of multiple ZIKV strains at a range of doses. Only a small percentage of mosquitoes were susceptible to infection orally (up to 10% for *Cx. pipiens*) and a range of 15–70% of intrathoracically inoculated mosquitoes demonstrated virus-positive bodies. However, unlike what was observed after IT inoculation of *Ae. aegypti* mosquitoes, there was no observed dissemination in any of the orally exposed *Culex* mosquitoes or salivary infection in any of the 38 intrathoracically inoculated mosquitoes showing body infection. The lack of detectable viral RNA or infectious virus in saliva, despite obviation of the midgut infection/escape barriers through IT inoculation, strongly suggests that ZIKV is incapable of infection of the salivary glands and/or entering the salivary fluids of *Cx. quinquefasciatus* and *Cx. pipiens*. Further, in vitro experiments demonstrate that ZIKV was unable to replicate in *Culex* cell lines.

The conclusions reported here regarding the inability of *Culex* mosquitoes to vector ZIKV correspond with a majority of recent studies addressing ZIKV transmission in *Culex* mosquitoes.^28^–^32^ In contrast, two accounts report differing ZIKV transmission potential of *Culex* mosquitoes. The first case is a summary of findings presented during a ZIKV workshop in Brazil in 2016 in which the authors report detection of virus from the salivary glands of *Cx. quinquefasciatus* at 7 and 15 days postexposure.^26^ To date, that presented data has not been formally published. However, a published study using several Brazilian ZIKV isolates with locally established *Culex* mosquito populations demonstrated incompetence of *Cx. quinquefasciatus* from Rio de Janeiro for infection, dissemination, and transmission.^30^ Guo and others orally exposed colonized *Cx. quinquefasciatus* from the Hainan Province of China with a low passage strain isolated from a traveler returning from Samoa (GenBank: KU866423). In this report, 8/10 (80%) saliva samples demonstrated the presence of ZIKV RNA by RT-PCR 8 days after exposure, with the ZIKV RNA positivity rate in saliva dropping at later time points.^27^ These results could indicate the possibility of differential susceptibility between Asian and American populations of *Cx. quinquefasciatus*. However, the incongruous comparison between detection of infectious virus, as traditionally measured by plaque formation or CPE assay, to RNA detection precludes drawing definitive conclusions between the two studies.

Phylogenetic analysis of mosquito-borne flaviviruses (MBFV) have long shown two distinct clades, one of which is primarily associated with *Aedes* mosquitoes with the other associated with *Culex* vectors.^40^,^41^ For instance, dengue-1, 2, 3, and 4 viruses are transmitted by *Ae. aegypti* mosquitoes, but not *Culex* mosquitoes.^42^ Considering the evolutionary precedent of MBFV steadfast adherence to being vectored by either *Aedes* or *Culex* mosquitoes but not both, it would be consistent that ZIKV would be vectored by *Aedes* mosquitoes. Similarly, *Culex*-borne flaviviruses typically use avian amplification hosts. Transmission of ZIKV to humans by *Culex* mosquitoes would require replication to high titers in avian hosts and potential epizootic spillover into humans as the *Culex* mosquitoes of concern primarily feed on avians and less frequently on humans. Blood meal analysis studies typically show humans to be a source of 0–2% of all blood meals taken by North American *Cx. pipiens*, *Cx. quinquefasciatus*, and *Cx. tarsalis* mosquitoes.^43^–^50^ Lack of suitable ZIKV avian hosts combined with low human blood feeding rates for *Culex* mosquitoes diminish the potential role of *Culex* mosquitoes as vectors regardless of their vector competence for ZIKV.

Our use of oral and IT exposure in *Cx. pipiens* and *Cx. quinquefasciatus* to show the inability of ZIKV to disseminate to the salivary glands in addition to the lack of observed ZIKV replication in *Culex* cell lines, strongly indicates that these North American *Culex* mosquitoes are not competent vectors of ZIKV. In conjunction with previously mentioned ecological requirements of utilizing *Culex* mosquitoes as vectors, we conclude that there is no evidence to support *Culex* mosquito monitoring or management with regard to ZIKV control efforts.

## Figures and Tables

**Table 1:** Infection, dissemination, and transmission rates after CPE analysis at 14 days postexposure in *Culex quinquefasciatus* and *Culex pipiens* orally exposed to infectious blood meals with strains of ZIKV

Species	Virus	*N*	Blood meal titer (PFU/mL) log_10_	Infection (%)	Dissemination (%)*
*Culex quinquefasciatus*	MR766	95	6	1 (1)	0 (0)
	PRVABC59	36	7.1	0 (0)	0 (0)
		48	5.9	0 (0)	0 (0)
		43	4	0 (0)	0 (0)
	R103451	35	7.6	0 (0)	0 (0)
		30	6.4	0 (0)	0 (0)
*Culex pipiens pipiens*	MR766	20	6	1 (5)	0 (0)
	PRVABC59	38	6	4 (10)	0 (0)

CPE = cytopathic effect; PFU = plaque-forming units; ZIKV = Zika virus. MR766 was isolated from Uganda in 1947, PRVABC59 was isolated from Puerto Rico in 2015, and R103451 was isolated from Honduras in 2016.

* Saliva samples not tested for transmission in the absence of dissemination.

**Table 2:** Detection of infection and transmission rates by CPE testing in *Culex quinquefasciatus* and *Culex pipiens* intrathoracically inoculated with ZIKV

Species	Virus	*N*	Inoculum stock (PFU/mL)log_10_	Infection (%)	Transmission (%)*
*Culex quinquefasciatus*	MR766	23	6.7	16 (70)	0 (0)
	PRVABC59	33	6	5 (15)	0 (0)
*Culex pipiens pipiens*	PRVABC59	28	6	17 (61)	0 (0)
*Aedes aegypti*	PRVABC59	12	6	12 (100)	8 (67)

CPE = cytopathic effect; PFU = plaque-forming units; ZIKV = Zika virus. MR766 was isolated from Uganda in 1947, PRVABC59 was isolated from Puerto Rico in 2015, and R103451 was isolated from Honduras in 2016.

* CPE-negative saliva samples were confirmed by reverse transcription polymerase chain reaction.
